# Is dogs’ tendency to follow human misleading communicative cues influenced by humans’ auditory perspective?

**DOI:** 10.1007/s10071-025-02028-y

**Published:** 2025-11-23

**Authors:** Lucrezia Lonardo, Victoria Berndl, Christoph J. Völter, Ludwig Huber

**Affiliations:** 1 Comparative Cognition, Messerli Research Institute, University of Veterinary Medicine Vienna, Medical University of Vienna and University of Vienna, Veterinärplatz 1, 1210 Vienna, Austria; 2 Department of Comparative Cultural Psychology, Max Planck Institute for Evolutionary Anthropology, 04103 Leipzig, Germany

**Keywords:** Theory of mind, Comparative psychology, False belief, Auditory perspective-taking, Altercentric bias, Animal cognition, Social cognition

## Abstract

Studying Theory of Mind in non-verbal populations requires designing tasks that address the distinction between responding based on directly perceivable cues and additionally inferring others’ mental states. We designed and pre-registered an auditory version of a non-verbal change-of-location task, to investigate whether dogs are sensitive to a human communicator’s mental states about the location of food. With control conditions we ruled out alternative cognitive processes such as associative learning. Dogs (*N* = 240) could witness that food was hidden first in one opaque bucket (A) and then relocated to a second opaque bucket (B) by an experimenter. Before being allowed to retrieve the food from one of the buckets, dogs received a misleading suggestion (A) from the communicator, who could not see the scene. In all conditions, the communicator could hear food being hidden in A, due to the presence of bells on the lid of this bucket. We manipulated whether she could also hear that food was removed from A and relocated to B (true belief) or not (silent bells on B, leading to her false belief). Importantly, in both conditions the communicator behaved identically (present in the room, suggesting A). Dogs’ responses were not statistically different from those of a previous study using a similar change-of-location task in the visual domain (Lonardo et al. *288*(1955), 2021, 10.1098/rspb.2021.0906). Dogs’ choices in the present study, however, did not differ significantly across conditions, suggesting that any auditory perspective-taking ability they might possess did not have a sufficiently large effect to be detected in this study.

**Supplementary Information:**

The online version contains supplementary material available at 10.1007/s10071-025-02028-y.

## Introduction

The expression “Theory of Mind” (ToM) was first adapted from the philosophical (Dennett 1978) into the empirical domain by primatologists (Premack and Woodruff 1978) and later on further elaborated by psychologists. According to the latter, we would define ToM as the cognitive ability to explain others’ behaviour on the basis of their minds: their knowledge, their beliefs and their desires, and knowing that when there is a conflict between belief and reality it is others’ belief, not the reality, that will determine their behaviour (Frith and Frith 2005). It is essentially the capacity to attribute mental states to others, which is crucial for social interaction and communication. A long-standing difficulty when studying ToM in non-verbal or pre-verbal populations has been that of designing tasks that can distinguish between the ability to only read an agent’s directly observable behaviour and the ability to additionally infer, from that behaviour, underlying, invisible mental states (Heyes 1998; Lurz and Krachun 2019). For example, a chimpanzee who approaches first food that, from a dominant conspecific’s perspective, is placed behind a barrier (Hare et al. 2001), might be mind-reading or behaviour-reading. The former implies attributing the mental state “(not) seeing” to the competitor. The latter implies having associatively learnt that dominant competitors who orient towards a food source with a direct line of gaze, in daylight and with open eyes are likely to obtain the food reward, so it is better to look for food elsewhere.

So far, methods that tapped into the visual modality only, mirrors (Lurz et al. 2018) and experience-projection methods (Kano et al. 2019; Karg et al. 2015; Lonardo et al. 2024; Meltzoff and Brooks 2008), have been employed to overcome the behaviour- vs. mind-reading difficulty with non- or pre-verbal populations. The advantage of experience-projection tasks is that they can equate behavioural cues between conditions and therefore they can only be passed by subjects who can attribute mental states (Heyes 1998). However, they place greater demands on the subjects than just mentalizing abilities. Indeed, they require the ability to project onto others the participants’ own egocentric past perspective.

To our knowledge, experience-projection tests have been adapted for dogs (*Canis familiaris*) in only two studies (Lonardo et al. 2024; West-Brownbill et al. 2024). In these, dogs were initially familiarised with the properties of a novel apparatus, that on one side allowed visual access, while on the other side it did not. Crucially, from the dogs’ starting position during subsequent test trials both sides looked identical (except for irrelevant features such as shape or colour) and dogs had to infer that the experimenter could have only seen their approach to forbidden food through one side of the apparatus but not the other. However, in neither study did the dogs show evidence of experience-projection; they did not preferentially approach the side of the apparatus from which they could have not been seen. One possibility to explain these results is that experience-projection tests, as they have been implemented so far, are too demanding for assessing dogs’ perspective-taking abilities, since they additionally require the ability to project onto others. Moreover, experience-projection tasks have been criticised because, while they do control for behaviour-reading, they do not necessarily allow to dissociate between seeing and line of gaze (Lurz and Krachun 2019). Indeed, in the self-experience phase, subjects may learn that the opaque barrier does not allow an uninterrupted line of gaze to objects behind it, in contrast to the transparent barrier. Based on this acquired information about the obstacles, subjects then infer in the test whether another individual has an unobstructed line of gaze or not (without necessarily having to reason about the mental state of “seeing”).

While mirrors have been proposed as a possible way to control for the line of gaze criticism, it is unclear what dogs understand of reflective surfaces. Hence, in the present study, we moved away from experience projection tasks but still addressed the distinction between the ability to mind-read and behaviour-read by using a novel paradigm in the auditory domain, following the logic of altercentric interference tasks. We hypothesised that dogs might be subject to an altercentric bias, i.e., that their cognition might be influenced by the presence and focus of attention of others (Kampis and Southgate 2020; Southgate 2020), given their well-documented sensitivity to human behaviour and attention (Huber and Lonardo 2023; for a review on dogs’ perspective-taking). Specifically, dogs have already been found to show sensitivity to different visual and auditory cues to humans’ perspectives in socio-cognitive tasks. For example, in a study by Bräuer et al. (2013) they preferred to approach forbidden food by walking on a silent rather than a noisy mat. Moreover, in a study by Kundey et al. (2010) both pet and shelter dogs preferred to steal forbidden food from a silent over a noisy container, but only if the experimenter was not looking in the direction of the containers. Further, dogs prefer stealing from behind opaque compared to transparent barriers (Bräuer et al. 2004; Call et al. 2003; Huber et al. 2025). However, in the absence of appropriate control conditions, these clever behaviours do not necessarily show that dogs are sensitive to others’ perspectives. Rather, dogs’ performance in these tasks might be guided by their own egocentric perspective: what the dogs themselves can or cannot potentially hear/see (Bräuer et al. 2013). Indeed, dogs in the previous studies might have just avoided the carpet that made a noise or the food that was close to a transparent barrier from which they themselves had previously seen an experimenter. Making a noise or seeing a human (not necessarily being heard or seen) when stealing might well be directly perceivable cues that pet dogs have learned are associated to a stealing failure and therefore should be avoided.

Because the literature on canine perspective-taking has rarely attempted to differentiate between these alternative explanations (with first exceptions being Lonardo et al. 2024; West-Brownbill et al. 2024), it is still unclear whether dogs’ abilities to successfully interact with us rely uniquely on their own egocentric perspective or whether dogs can additionally take into account others’ mental states. In the present study, we set out to answer this question by adapting the change-of-location task reported in Lonardo et al. (2021) for the auditory domain.

Closely following the procedure of Lonardo et al. (2021), also in the present study dogs were familiarised to the possibility of obtaining food from one of two opaque plastic buckets after witnessing its baiting. After having seen in which bucket the food was placed and before they were allowed to make their choice between the buckets, however, dogs received a misleading suggestion from a human communicator. The communicator suggested to the dog the empty bucket. Following the change of location paradigm (Wimmer and Perner 1983), in all test conditions one experimenter hid the treat in one bucket first (A) and then relocated it to a second bucket (B), in full view of the dog (Fig. 2). The communicator could always hear that a treat had been hidden in bucket A, due to the presence of bells on the lid of the bucket and due to the treat being audibly dropped on the bottom of the bucket. We manipulated the communicator’s apparent mental state by varying whether she could also hear that bucket A was opened again and the treat was relocated to bucket B (True Belief condition) or not (False belief condition). The crucial difference of the present study, compared to Lonardo et al. (2021), was that here in the True and False Belief conditions, we restricted the communicator’s perception of the food hiding events to the auditory domain (the communicator closely faced a wall of the room, with her back turned towards the hiding scene), which prevents the dogs from assessing the human’s attentional state from any directly observable behavioural cue. The True Belief communicator could hear the treat being removed from bucket A and relocated to bucket B due to the presence of bells on the lid of the buckets. In contrast, the False Belief communicator could not hear the translocation due to the removal of the clappers from the bells on the lid of bucket B. To control whether the different auditory cues were sufficient to elicit a differential response between True Belief and False Belief conditions, we designed a control condition in which the communicator had visual access to the scene, and therefore was knowledgeable about the food relocation, but heard exactly the same auditory cues as in the False Belief condition (Knowledge Sound Control condition).

Based on previous literature (e.g., Barnard et al. 2019; Chijiiwa et al. 2021; Marshall-Pescini et al. 2011; Prato-Previde et al. 2008; Szetei et al. 2003), we expected dogs to be sometimes misled into choosing the bucket they knew to be empty. Dogs often prioritize human social actions over conflicting physical cues. For example, Chijiiwa et al. (2021) showed that dogs were more likely to choose a now-empty container from which a human had just removed food, rather than another container that still contained food. These findings highlight how dogs may be biased to interpret human actions as communicative or meaningful, even when they conflict with environmental information. Indeed, in a previous study using this change of location task in the visual domain (Lonardo et al. 2021), we found 38.5% of the dogs choosing the empty, suggested bucket. However, this choice was modulated by the informant’s mental state. Specifically, more dogs in the False Belief condition followed the communicator’s suggestion, a finding that is similar to those later on found with chimpanzees (Lurz et al. 2022) and human infants (Kampis and Kovács 2022; Mascaro and Kovács 2022) tested on implicit “altercentric interference” tasks. In such tasks, participants’ behaviour is implicitly biased by the (incongruent) perspective of an incidentally present experimenter, even when the experimenter’s perspective is actually irrelevant to the participants’ task (e.g., making a quantity judgement, finding a hidden reward). Such findings, at least in tasks with humans, are interpreted as evidence of the participants implicitly and spontaneously taking on the experimenter’s perspective (Kampis and Southgate 2020; Rakoczy 2022). For example, knowledgeable children searched longer in a box when an experimenter believed it contained a reward, and chimpanzees searched for hidden food closer to where a human experimenter falsely believed the reward was hidden. Similarly, if the dogs’ choices are automatically biased by the communicator’s mental state also in our task, we would predict that more dogs choose the empty bucket when the suggestion came from a communicator who actually believed the bucket to be baited (False Belief) compared to when the suggestion came from a communicator who, like the dogs, knew the suggested bucket to be empty (True Belief). As explained above, this altercentric modulation of dogs’ choices (being more misled into searching an empty bucket when a human falsely believes it to be baited) can happen even though the communicator’s suggestion is completely superfluous for the dogs to achieve their goal, which we assume is to obtain the food. For the Knowledge Sound Control condition, we predicted that if dogs’ choices are driven purely by the noises made by the lids of the buckets, they should have followed the communicator’s misleading suggestion at similar rates between the False Belief and the Knowledge Sound Control condition, because in these two conditions the auditory cues were identical. If, instead, dogs’ choices are driven by the communicator’s mental state, we would predict a similar number of dogs following the communicator’s misleading suggestion between the True Belief and the Knowledge Sound Control condition, because in both these conditions the communicator ends up knowing or believing that food is in bucket B (see Table 1 for a summary of the hypotheses and predictions).

**Table 1:** H0 (null hypothesis): dogs are not sensitive to the difference between human TB and FB, when these are induced through auditory cues, in the absence of directly observable behavioural differences between conditions. H1 (mind-reading): dogs are sensitive to the difference between human TB and FB, when these are induced through auditory cues, in the absence of directly observable behavioural differences between conditions. Our prediction that dogs should follow the communicator’s suggestion (and hence choose the empty bucket A) more often in the FB than in the other two conditions is based on a hypothesised implicit altercentric interference effect. Indeed, only in the FB condition, but not in the other two conditions, the communicator’s belief, however irrelevant for the dogs to find the food, is incongruent with the dogs’ own belief and this should be sufficient to make dogs automatically more error-prone (assuming that dogs’ goal is to obtain the food, which they believe to be in bucket B). H2: dogs respond based on an egocentric strategy and associative learning in this task.

Hypothesis	Prediction about bucket A choices
H0 (null)	FB = TB
H1 (mind-reading)	FB > TB = KSC
H2 (egocentric strategy, associative learning)	FB = KSC

Finally, because there is evidence that experience in dog sports (e.g., Heberlein and Turner 2017; Marshall-Pescini et al. 2009) and breed type (e.g., Gácsi et al. 2009; Heberlein and Turner 2017) can influence dogs’ performance in socio-cognitive tasks, we also explored the effect of these variables in our task

## Materials and methods

The study was discussed and approved (ETK-067/06/2024) by the ethics and animal welfare committee of the University of Veterinary Medicine of Vienna in accordance with GSP guidelines and national legislation. Written consent to participate in the study was obtained by the dogs’ owners. The hypotheses, predictions, experimental design and data analysis plan were pre-registered prior to data collection at the Open Science Framework (https://osf.io/u7jer/?view_only=6dd3dd136d7a43f082acef82a4d4bf03).

### Subjects

The final sample consisted of 240 purebred dogs (130 females) belonging to 60 different breeds and classifiable under 7 different FCI groups. The age range went from 6 months to 12.9 years; the mean age was approx. 5 years. The data file “tested_dogs.csv”, available in the “data” subfolder of the repository: https://github.com/lonardol/auditory_false_belief_dogs, reports information on the demographics, counterbalancing and individual performance of each tested dog. In addition, 125 more dogs came to the lab but were not tested due to either failing to meet our inclusion criteria in one of the familiarisation phases (*N* = 110); an experimenter’s mistake (*N* = 1); being too shy or not food motivated enough to try or to succeed in opening the buckets independently at any point during the experiment (*N* = 11); being too aggressive towards unfamiliar humans (*N* = 2) or deaf (*N* = 1). Additionally 6 more dogs were tested but excluded from analyses due to either not making a choice in the test trial (*N* = 1); very loud vocalisations that made it unclear if the dog could hear the bells noise during the test trial (*N* = 1); an experimenter’s mistake (*N* = 2) and exceeding our pre-registered sample size on the final data collection day, when it was too late to cancel the appointment with the dogs’ caregivers (*N* = 2).

### Set up

**Fig. 1:**
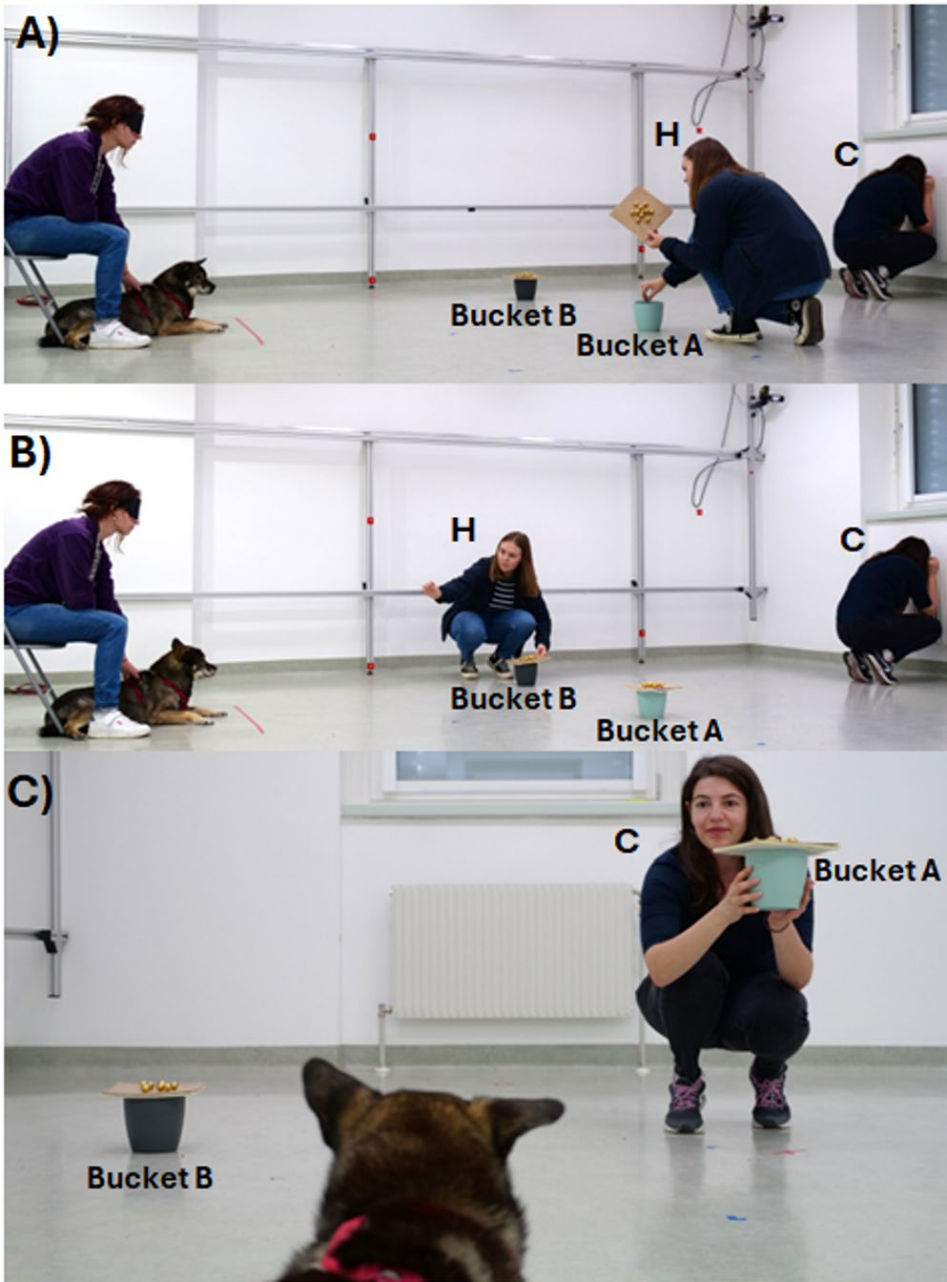
Experimental procedure. (**A**) The hider hides the treat in bucket (**A**) H stands for hider and C for communicator. (**B**) the hider relocates the treat to bucket (**B**). (**C**) The communicator suggests bucket A. These three phases were present in all experimental conditions. The dog could always see the whereabouts of the treat. The blindfolded owners sat on a chair while holding their dog (pictures **A** and **B**). The dog shown in the picture is not one of the participants

All experiments were conducted in the same 605 × 725 cm room of the Clever Dog Lab, Vienna (Figure S1). At the beginning of each trial, dogs were held in a position equidistant from both buckets by their owner, seated on a chair behind the dog. The performance of dogs in all trials (familiarisation and test phase) was recorded with a four-camera system. For most dogs we used two 12.5 cm high opaque plastic buckets (flowerpots, diameter: 13.7 cm) of different colours (one blue, the other grey), positioned at 1.44 m from each other and equidistant to the dog (1.81 m, see Fig. 1 and S1). For shorter or shyer dogs, we used a smaller set of buckets (height: 10.5 cm, diameter: 12.2 cm), one brown, the other green, and for bigger dogs a larger pair of buckets (height: 15.5, diameter: 18.2), one blue, the other brown. Each pair of buckets was covered with an opaque, cardboard lid (side: 20 cm, thickness: 0.3 cm), to which 10 bells were attached. The bottom of the lid was covered in a subtle rubber foam layer (0.7 cm thick), to avoid that closing the lid on the buckets would make any noise. Dogs were shown before the beginning of the experiment how to push the lid off the buckets with snout or paw. Before each session, the inside of both buckets was dusted with the same type of food that was used in the experiment (see SM for more details). Water was available ad libitum.

### Design and procedure

In a between-subjects design, each dog was tested in a single test trial of only one of the three experimental groups. All experiments took place during a single session of approximately 30 min. They consisted of the three familiarisation phases and the subsequent test trial; each experimental phase was performed immediately after the other, without any intervals in between. From the beginning of the first familiarisation phase, owners were asked to wear a blindfold during each trial to avoid inadvertently giving directional cues to their dog with their gaze (Fig. 1). The familiarisation aimed at introducing the dog to the set-up, the experimenters and the possibility of retrieving food from one bucket per trial. We also used the familiarisation as a screening to assess whether each dog intuitively understood visible displacements and was willing to follow the communicator’s signal when prevented from witnessing where food had been hidden (see Supplementary Material for more details).

Each dog was familiarised to the experimental procedure in three subsequent steps. In the first familiarisation phase we showed to the dogs an experimenter (the hider) hiding food in bucket A first and sometimes relocating it to bucket B. The goal of this phase was to exclude dogs that did not understand the visible displacement. In the second familiarisation phase, dogs started outside of the experimental room and could therefore not witness the hiding of the treat. Upon entering the room, however, they found another experimenter (the communicator) crouching down and facing the wall, with her back turned to the dog and the buckets. Once the dog and owner had moved into their predetermined starting position, the communicator would turn around and suggest to the dog the correct location of food (counterbalanced). The goal of this phase was to only include dogs being ready to follow the communicator’s suggestion in the absence of own knowledge. Finally, the third familiarisation phase combined the previous two in that dogs could see both the hiding of the treat performed by the hider and a correct suggestion from the communicator. The goal of this phase was to show dogs that even though the communicator was facing away from the hiding scene, she would still interpret correctly the noises of the bells and would suggest the baited bucket both in a trial with the treat relocated from A to B and in a trial without relocation.

By the end of the familiarisation, dogs were accustomed to a series of events. The hider always hid food in one bucket first (bucket A; Fig. 1A). When hiding food in one bucket, the hider opened the lid (with a bells noise), dropped the food audibly in it and closed the lid again (second bells noise). In half of the familiarisation trials, the hider additionally opened bucket A again (third bells noise) and visibly (for the dogs) transferred the food to a second bucket (B; Fig. 1B) before leaving the room. Additional auditory cues produced by the hider during the food relocation were: footsteps while walking from A to B, opening of bucket B (first bells noise close to B), dropping the food audibly in B and closing the lid on bucket B (second bells noise close to B). The communicator always faced a wall, with her back to the dogs and the buckets. Therefore, during the familiarisation, her knowledge about the food location could be inferred from the auditory cues described above. During all familiarisation phases, the communicator would suggest to the dogs the baited bucket by turning around, approaching the baited bucket, picking it up, alternating gaze between it and the dog while uttering the sentence “look, this is good, this is very good”, (in English, to avoid using commands known to the dogs) with a positive voice intonation. Then, she would put the bucket back to its original position on the floor, walk back to her starting position, equidistant from both buckets, this time facing the dog but looking to the floor and she would say “OK” to signal to the blindfolded owner to release the dog. During all familiarisation trials involving the communicator, she always suggested to the dogs the correct (baited) bucket (in trials without translocation, A, in trials with translocation, B).

**Fig. 2:**
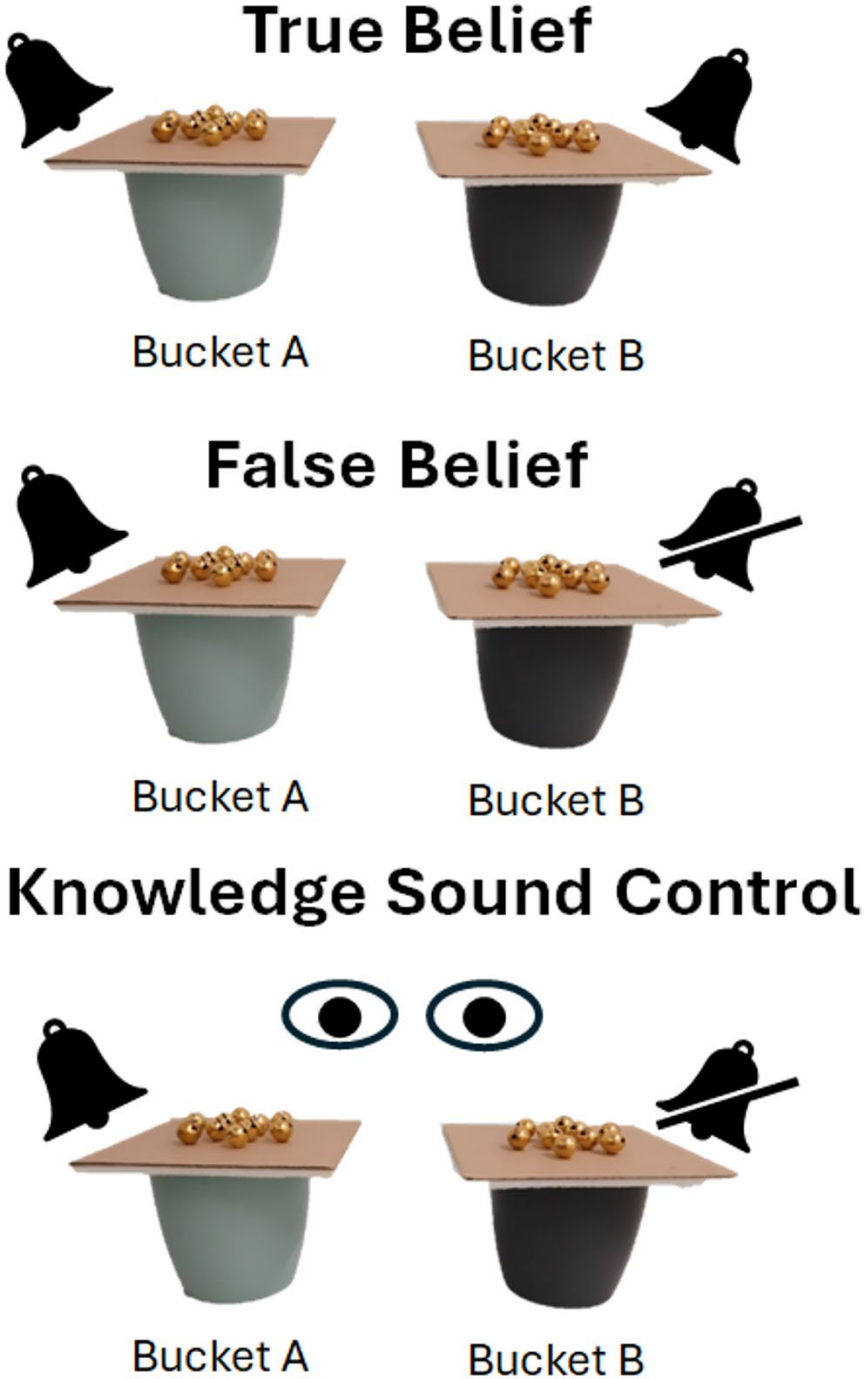
Schematic representation of the three experimental conditions. The colour of bucket A was counterbalanced between dogs

After the familiarisation phases (identical for all tested dogs) we divided subjects into three equally sized experimental groups: False Belief (FB), True Belief (TB) and Knowledge Sound Control (KSC). During test trials of all conditions, for the first and only time, did the communicator suggest the empty bucket A (Fig. 1C). Importantly, the communicator’s suggestion during test trials did not differ between the experimental groups (Fig. 2). Dogs did not need this suggestion to find the food because in all conditions they witnessed directly the full hiding sequence.

In the TB Condition, the events unfolded just as described for the final familiarisation phase, the only difference being that the communicator suggested bucket A despite this being empty after the translocation. Namely, the hider would open bucket A (first bells noise), drop food in bucket A (food noise), close bucket A (second bells noise), open bucket A again (third bells noise), remove the food from there and close bucket A (fourth bells noise). Then, she would walk to bucket B (footsteps), open its lid (first bells noise close to B), drop the food in B (food noise) and finally close B (second bells noise close to B) before leaving the room. Therefore, the communicator was left with the true belief that food was in B.

In the FB condition, we replaced the lid on bucket B with one that looked identical (cardboard with bells attached to it) but that was silent even when moved, because the clappers of all bells had been removed. Additionally, we placed on the bottom of bucket B a 1 cm-thick padding to ensure that the food being dropped in bucket B would not make any noise. Hence, the auditory cues heard by the communicator (who, like in the TB condition, kept facing the wall, with her back to the buckets) in the FB condition were: opening of bucket A (first bells noise), food being dropped in bucket A (food noise) and closing of bucket A (second bells noise), just like in the TB condition. All additional auditory cues that characterised the TB condition were made silent in the FB and SKC condition: the hider would open bucket A again but this time very carefully to ensure no noise was produced (no third bells noise), remove the food from there and close bucket A silently (no fourth bells noise). Then, she would walk to bucket B silently (shoes were taken off prior to the start of the test in the FB and KSC conditions), open its silent lid (no bells noise close to B), drop the food in B (no food noise due to the padding) and finally close B (no second bells noise close to B) before leaving the room. The auditory cues were identical between FB and KSC condition, but in the latter the communicator was turned towards the buckets. Therefore, she could see the whole hiding sequence and followed it attentively. By contrast, in the FB and TB conditions the communicator could not see any part of the hiding. Examples of actual experimental video recordings of test trials for each condition are available at: https://github.com/lonardol/auditory_false_belief_dogs in the “videos” subfolder.

At the end of each hiding sequence of test trials only, the hider also took care to sneakily remove the treat from bucket B after dropping it in there and before closing the lid. The removal of food was not noticeable for the dogs but it was an additional way of controlling for odour cues, to ensure dogs could not make a choice based on their sense of smell but only based on what they had observed, a common practice in socio-cognitive tasks with dogs.

The blindfolded owners could probably still differentiate whether bells or food noises were coming from their left or right side, just like the communicators could. However, to the owners, these sounds only signalled that one or the other bucket were being opened or closed but the blindfold still prevented them from knowing in which bucket food was being left or removed from (if any). Owners were only debriefed about the exact study procedure after the end of the test, and until that point did not even know that (only) one piece of food was being hidden per trial, let alone where. We did not assume that the blindfolded owners’ inadvertent reactions to sounds might have influenced the dogs’ choices in this study for different reasons. First, there is evidence that dog owners do not influence their dogs’ choices through subtle cues in a two-choice task comparable to ours (Schmidjell et al. 2012). Second, the same study showed that an experimenter’s communicative cue can override even owners’ overt directional cueing. However, in the present study owners were obviously instructed to avoid such cues and to not interact with their dogs in any way during trials, other than opening their hand to release their dog when they heard a signal from the experimenters. Nevertheless, future research adopting the same paradigm might ask the owners to wear noise-cancelling headphones and communicate to them in a different way when it is time to release their dog.

The sample was counterbalanced for age, sex, experimenters’ identities and breed across experimental groups as much as possible (see Table S1). All dogs were tested with communicators and hiders unfamiliar to them. Across dogs, to control for any biases or preferences for individual experimenters, four different experimenters took the communicator and hider roles. We perfectly counterbalanced across dogs the experimental condition, the (left-right) position of bucket A relative to the dog, the type of trial presented first in familiarisation phase 2 and in familiarisation phase 3 (relocation or no relocation first; see Supplementary Materials).

### Power analysis

To determine the sample size, we had conducted a power simulation before registering our study using Monte Carlo simulations. We simulated 1000 datasets following the design described in Sect. 1.3 (between-subjects design, with each individual assigned to only 1 test condition and tested in a single trial). For each dataset, we parameterized the choice response, age, sex, breed group and first baited bucket for a number of subjects varying from 120 to 240, with increments of 12. Based on the data in Lonardo et al. (2021), we simulated on average 50% of the dogs in the FB condition to choose bucket A and 25% of the dogs in both the TB and KSC conditions to choose bucket A, by repeatedly sampling the response from a binomial distribution. We simulated the random slope of condition to be 1 and the random intercept of breed group to be 0.001. With every simulated dataset, we fitted a full binomial GLMM as the one described in Sect. 1.5 and a null model that was identical to the full model but lacked the fixed effect and random slope of condition. We compared the full to the null model with a likelihood ratio test, implemented using the R function “anova”. The results indicated that with a total of 240 dogs (80 dogs per condition), 82% of the models converged and were significant when compared to the respective null model (likelihood ratio tests: *p* < .05). With 240 dogs, the average simulated response (choice of bucket A) was 48.6% in the false belief condition, 28.8% in the knowledge condition and 28.4% in the knowledge sound control condition. This means that with 80 dogs per condition we should have sufficient power to detect an effect comparable to the one reported by Lonardo et al. (2021), who found a 19% difference between true and false belief conditions.

### Scoring and analyses

From the video recordings of the test trials, we scored dogs’ first choice, i.e., whether dogs touched first (with their nose or one of the front paws) bucket A, as suggested by the communicator, or bucket B, where dogs saw food being hidden last. All statistical analyses were performed in R (R Core Team 2022; version 4.2.2). To assess, inter-observer reliability, two coders independently scored the dogs’ choice from 25% of the videos (60 subjects). Their inter-observer agreement was almost perfect (percentage agreement = 96.7%; Cohen’s Kappa = 0.93; z = 7.25. *P* < .001).

To test whether the number of dogs choosing bucket A (scored as 1) or B (scored as 0) differed between the three conditions, we used a Generalised Linear Mixed Model (GLMM 01; Baayen 2008) with binomial error distribution and logit link function. We included as sole test predictor with fixed effect condition (KSC, FB and TB). Control predictors with fixed effect were the dogs’ age (in months, z-transformed), sex and first baited bucket (on the right or left side of the dog). We also included the random slopes of condition, sex, first baited bucket (all manually dummy coded and centred) and age (z-transformed) within the random intercept of breed, as for most of these we had repeated observations within breed.

In addition to our pre-registered analysis, we also explored potential breed type differences (cooperative vs. independent; classified similarly to previous studies: Gácsi et al. 2009; Heberlein and Turner et al. 2017; Lonardo et al. 2021), since our sample also included a small sub-sample (*N* = 30) of independent breeds. To this end, we fitted the same GLMM described above, but including as sole test predictor the interaction between breed type and condition (GLMM 02). Moreover, we explored whether having received professional training, for example due to participation in dog sports, affected the dogs’ choices (the dogs that had received professional training in our sample were 180). To this end, we fitted again the same model, but including the interaction between having received professional training (yes/no) and condition (GLMM 03). Finally, we decided to compare quantitatively the results of the present study to those of Lonardo et al. (2021)’s study (GLMM 04). For this exploratory analysis, we used 180 observations (from as many dogs) from the original Experiments 1 and 2 and the 240 observations of the present study (in total, 420 observations). GLMM04 was identical to GLMM01 with the addition of the fixed effect and random slope of study (factor with 2 levels: 2021 or 2025) and their interaction with condition (factor with 3 levels: TB, FB or Control, C). Since ten dogs took part in both studies, we additionally included a random intercept for subject ID. Pairwise comparisons between the three levels of condition were conducted with the R function *emmeans* of the homonymous package (Lenth 2023), correcting the p-values with Tukey’s method.

In addition to the frequentist analyses, we also conducted an exploratory Bayesian model comparison to further examine whether dogs’ choices differed between the three conditions. Two Bayesian logistic regression models were fitted using the brms package (Bürkner 2017): a null model including only the intercept and control predictors (z-transformed age, sex, and first baited box), and a full model with the condition predictor in addition to the same control predictors. Regularizing priors were applied: N(0, 1.5) for the intercept and condition parameters (which place approximately 95% of prior probability within the bounds corresponding to response probabilities between 0.05 and 0.95), N(0, 0.75) for binary predictors (sex and first baited box), and a narrower N(0, 0.1875) prior for age (z-transformed). Both models were run with 5000 iterations across 8 chains to ensure robust Bayes factor estimation. More details on the analyses are in the Supplementary Materials.

## Results

Overall, across the three experimental groups (FB, TB, KSC), significantly more dogs (58%) chose the bucket where they last saw the treat being hidden, rather than following the communicator’s misleading suggestion (not pre-registered binomial test, p-value = 0.017).

**Fig. 3:**
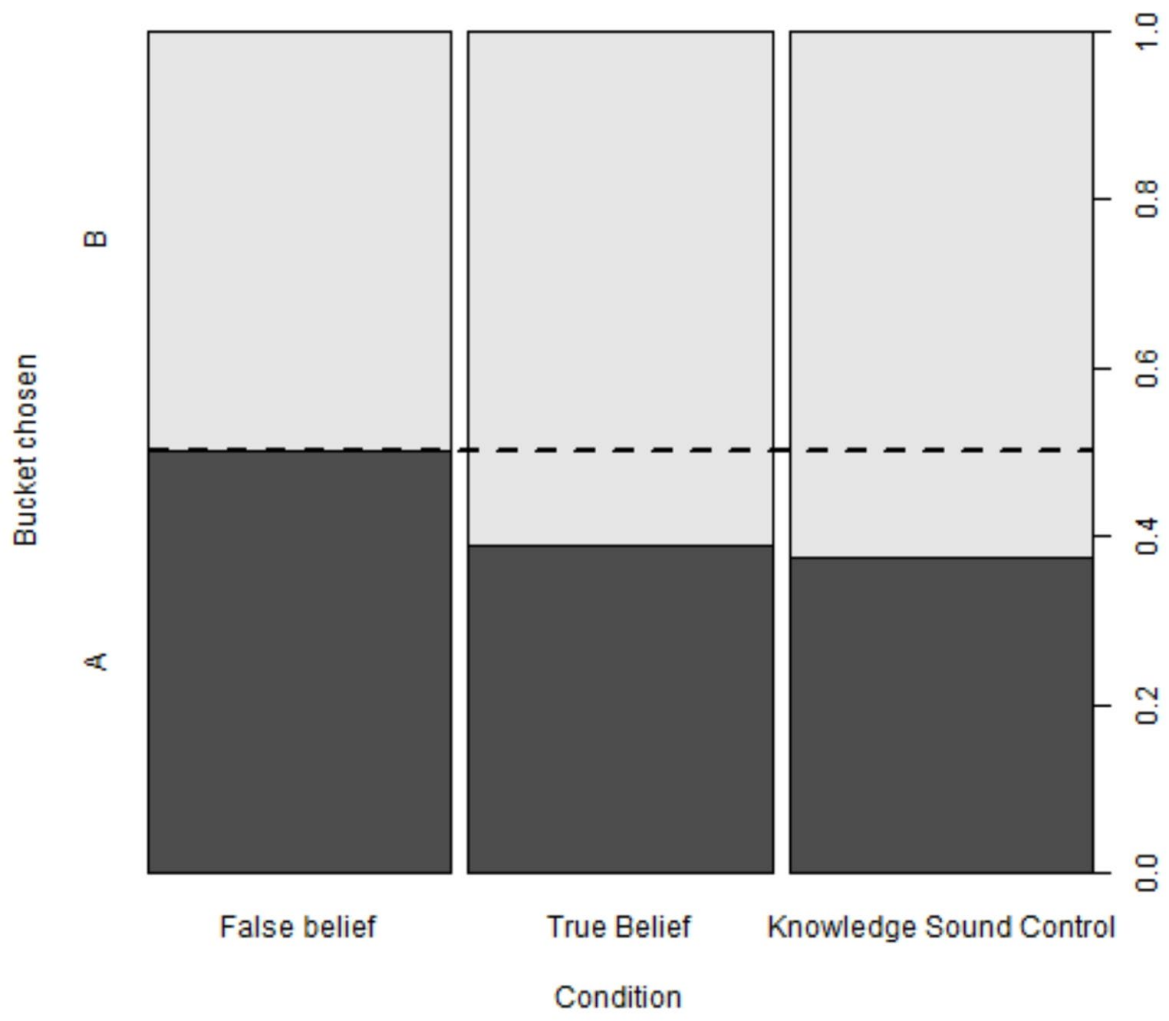
Mosaic plot showing the proportion of dogs choosing the suggested but empty bucket A (dark grey) and B (light grey) in each condition (*N* = 80 per condition). The dashed line indicates chance level (0.5). Dogs’ performance did not statistically differ between conditions (see Table S2)

However, dogs’ likelihood to choose bucket A did not significantly differ across the three experimental groups (likelihood ratio test of condition, binomial GLMM 01: χ^2^ = 3.03, df = 2, *p* = .220). Dogs’ rate of choosing bucket A was 50% in the FB condition, 39% in the TB condition and 38% in the KSC condition (Fig. 3). Dogs’ choice of bucket B in the TB condition approaches significance (not pre-registered binomial tests: *p* = .057) and dogs’ choice of bucket B in the KSC condition is significantly above chance level (*p* = .033), suggesting that dogs in the KSC condition avoided the communicator’s misleading suggestion, unlike in the FB condition. To exclude that dogs in this study had a special affinity for bucket A or did not understand the task as expected, we explored their performance also in the third familiarisation phase, in which the communicator had a true belief about the final location of food and suggested bucket B. In this case, only approx. 4% of the tested dogs chose bucket A on their first relocation trial.

When evaluating the effect of breed type and training experience on dogs’ choices, because neither of the likelihood ratio tests of the interactions were significant (interaction between breed type and condition: χ^2^ = 1.85, df = 2, *p* = .396; interaction between training experience and condition: χ^2^ = 0.38, df = 2, *p* = .828), we removed them from the models and retained only the fixed effects, again considering only condition as test predictor. Dogs’ choices were not affected by their breed type (cooperative vs. independent; likelihood ratio test of breed type, binomial GLMM 02: χ^2^ = 0.07, df = 1, *p* = .794), nor by their training experience (likelihood ratio test of training experience, binomial GLMM 03: χ^2^ = 2.13, df = 1, *p* = .145).

When comparing the present results to those obtained in the visual task (Lonardo et al. 2021), the effect of condition on the dogs’ choices did not vary significantly between experiments (interaction likelihood ratio test: χ^2^ = 1.04, df = 2, *p* = .594), so we removed the interaction from the model but retained both the fixed effect of condition and study. We found that dogs’ choices were not significantly different between the two studies (likelihood ratio test of study: χ^2^ = 1.90, df = 1, *p* = .169), but they did vary significantly between conditions (χ^2^ = 9.454, df = 2, *p* = .009). Specifically, in both studies, dogs chose the suggested but empty bucket A in the FB more than in the TB conditions and more than in the C conditions (FB-TB: z= −2.40, p_Tukey_= 0.044; FB-C: z= −2.84, p_Tukey_= 0.013). Moreover, in both studies dogs’ choices did not differ significantly between TB and C conditions (in both studies, a control condition in which the communicator was knowledgeable about the food final location; C-TB: z = 0.46, p_Tukey_= 0.891).

The results of the three binomial GLMMs investigating the effect of condition, breed type and experience with professional dog training on dogs’ choice are reported in detail in Supplementary Tables S2 to S4 respectively. The results of GLMM 04, comparing quantitatively the results of the present study with those of Lonardo et al. (2021), are reported in Table S5.

The Bayesian analysis corroborated the frequentist findings. Model comparison using Bayes factors (on log scale) yielded a value of 1.73 in favour of the null model. This represents extremely weak evidence for the null, indicating that both models accounted for the data approximately equally well. Nonetheless, inspection of the full model’s posterior estimates revealed a pattern consistent with the hypothesized effect: the predicted probability of choosing bucket A was highest in the false belief (FB) condition (M = 0.495, 95% CI [0.388, 0.601]), and lower in both the knowledge sound control (KSC; M = 0.373, 95% CI [0.275, 0.479]) and true belief (TB) conditions (M = 0.385, 95% CI [0.287, 0.493]). Posterior contrasts indicated that the FB condition was modestly higher than KSC (M = 0.121, 95% CI [–0.028, 0.270]), while the TB and KSC conditions were nearly identical (M = 0.012, 95% CI [–0.135, 0.158]). Importantly, the posterior probability that the FB condition had the highest bucket-A choice rate was 0.88, providing strong directional evidence in favor of the prediction that dogs were most likely to choose the suggested bucket in the FB condition. While the specific pattern observed aligns with our predictions, the overall evidence for a condition effect (based on the full-null model comparison) remains weak.

## Discussion

The aim of this study was to investigate whether dogs’ bias to follow a human misleading suggestion may be explained by implicit auditory altercentric perspective taking. We had predicted that dogs’ choices would differ between TB and FB conditions. Specifically, we expected that dogs should be more inclined to follow a human informant’s misleading suggestion to look for food in an empty bucket when the informant believed (wrongly) the bucket to be baited compared to when the informant believed the suggested bucket to be empty, based on a previous study using a similar paradigm in the visual domain (Lonardo et al. 2021). While the results went in the expected direction (Fig. 3), the same direction as in Lonardo et al. (2021), and the Bayesian analysis provided some support for the notion that dogs may be particularly likely to follow communicative cues in the FB condition, the effect of condition did not reach statistical significance in our preregistered confirmatory analysis. The comparison of the present findings to those of Lonardo et al. (2021) suggests that the effect of condition does not differ significantly between experiments and that the manipulation when pooling the data of two experiments is still significant. Despite the lack of a significant interaction between experiment and condition, the effect size in the current task might be smaller when compared to the visual version of the same change-of-location task. The smaller effect size might be due to a selective difficulty in auditory perspective-taking, to a difficulty in interpreting the attentional state of a back-turned human, or to a difficulty in disengaging from an egocentric strategy based on what the dogs themselves could perceive (in addition to other possible task- and statistical power-related factors discussed below).

Overall, across all three experimental conditions, more dogs chose the bucket where they last saw the treat being hidden, rather than following the communicator’s cue. This suggests that the exclusion criteria of our familiarisation phases were effective in only retaining dogs with an understanding of the visible food displacement. It thus makes it unlikely that those dogs that chose bucket A did so due to a memory failure. Our inclusion criteria might also explain why in the present study we do not find that dogs are so susceptible to the A-not-B error as in previous studies (e.g., Topál et al. 2009).

Associative learning seems unlikely to explain results in this task, as dogs could not differentiate between conditions that provided different auditory cues (KSC and TB), suggesting that such cues are not sufficient for dogs to pass this task. In a similar vein, neither could it be hypothesised that dogs preferred the bucket that was silent (bucket B in the FB and KSC conditions): dogs chose bucket B 50% of the times in the FB condition and they chose the silent bucket B at a similar rate (62%) in the KSC as the audible bucket B in the TB condition (61%), suggesting that their choices were mainly driven by their witnessing of the last baiting of bucket B.

At the same time, however, dogs’ failure to differentiate significantly between TB and FB conditions suggests that they might not be sensitive (enough) to others’ auditory perspective, or that our task was too cognitively demanding or not suitable to highlight this ability in dogs. If the first explanation is true, dogs’ successful performance in previous perspective-taking tasks using the stealing paradigm might also be explained in terms of their adopting an egocentric strategy to solve the task. Consistent with the hypothesis that dogs might not be capable of de-centring from their own perspective is their performance in the studies employing experience-projection methods (Lonardo et al. 2024; West-Brownbill et al. 2024). In these, dogs could not project their own previous experience with novel barriers onto the experimenters.

The innovation of the present task relative to most previous ones is that the expected pattern of results cannot be obtained if participants use an egocentric strategy, i.e., based only on the sounds the dogs themselves hear, given that the auditory cues are equated between FB and KSC condition. To discriminate between our conditions, subjects must consider (or be automatically influenced by) the communicator’s perceptual access to the information, i.e., this task requires altercentric perspective-taking. However, dogs in the current implementation of the task failed to significantly discriminate between conditions. While in comparison to the present study in Lonardo et al. (2021) the proportion of FB dogs following the suggested, empty bucket A was quite similar (48% vs. 50%), the proportion of TB dogs was lower (29% instead of 39%). This difference could possibly be explained by the fact that, contrary to the previous study adopting the change-of-location task in the visual domain, in the current implementation of the task, dogs could not rely on behaviour-reading to differentiate between conditions. While in the study by Lonardo et al. (2021) the TB and FB communicators behaved differently, namely being present or absent in the room at the moment of the food relocation from bucket A to B, in the present study the communicators behaved identically between TB and FB condition (being always present in the room and facing the wall). Hence, dogs’ failure to differentiate between conditions in the present study might indicate that at least some of them rely primarily on behaviour-reading to skilfully interact with us. Indeed, when the experimenters behaved identically between TB and FB conditions, by always facing the wall, dogs did not react differentially between conditions (at least not with the same effect size as in the visual task) even though these identical behaviours were supposedly characterised by different mental states (TB and FB).

An alternative explanation of the small, not significant difference between TB and FB conditions is that dogs might have failed to infer that a back-turned human is still paying attention to events happening behind her or that the human’s hearing abilities are good enough to track the hiding events from the bell sounds. In reality, we can confirm that the communicator was listening and could track the hiding events that were associated with the bells. Moreover, due to the familiarisation phases, the dogs could assume that the communicator was keeping track of the food whereabouts auditorily, since, during the familiarisation, the communicator always correctly suggested the baited bucket based on auditory cues alone. Further, to explain the lack of a significant effect of condition, it could be hypothesised that (some) dogs in the FB condition might have attributed knowledge to the communicator because the duration of the delay after the first hiding event (the period of silence following the hiding in A) was roughly the same as in familiarisation trials with reward displacement. However, it is important to note that during our experiment (including the familiarisation) dogs in the FB condition never saw the communicator react as if she was aware of the food relocation in the absence of any sounds from bucket B. In contrast to looking, which is accompanied by remotely perceivable behaviours such as open eyes, head or even body orientation, whether a human is listening or not is per se not perceivable by others. Others can notice the auditory perception of agents only if they react to auditory signals, e.g. a sudden noise, with head or body movements. But the communicators in the present study have purposefully avoided such visible indications of their hearing. Thus, we need to consider that our communicators behaved in a quite strange manner. They were facing the wall motionless and did not react in any way to what was going on behind them until the moment of their suggestion. This strange behaviour might indicate that the communicator is not paying attention or not interested in what is going on in the room. However, since dogs have everyday experience with humans being exposed to sounds without necessarily showing overt behavioural responses to what they hear, we argue that the ecological validity of this task is still greater than the one of experience-projection methods (Lonardo et al. 2024; West-Brownbill et al. 2024), in which dogs have to learn the properties of novel visual barriers. Nevertheless, it remains unclear (and it was part of our research question) whether dogs attribute hearing (and, as a consequence, maybe also knowledge and beliefs) to humans merely based on exposure to sounds without any behavioural reaction on the part of the human.

The possibility that dogs might implicitly take into account the communicator’s auditory perspective but may be still misled by the communicator’s suggestion is not likely, because our task pits these two pieces of information against each other. The mental state (e.g., TB/FB) is different between conditions while the suggestion (bucket A) remains the same. If dogs are sensitive to the difference between mental states, they should react differently to these conditions (due to an altercentric bias: in the FB condition, but not in the other two conditions, the communicator’s perspective is incongruent with the dogs’ and this should make the dogs more error-prone). If dogs are not sensitive to the difference between mental states, they should respond identically between conditions as they witness the same misleading suggestion in all conditions.

Future research could assess the performance of guide dogs in perspective-taking tasks, as their training and experience might have made them more skilled at reading people’s visual and auditory perception. For example, more guide dogs than pet dogs learned to ask for inaccessible food by producing an auditory cue (Gaunet 2008), even though Gaunet (2010) found no difference between guide and pet dogs requesting a toy instead of food.

Another explanation for the ambiguous findings is a lack of statistical power. We determined the current sample size using a power simulation based on the findings of Lonardo et al. (2021) and added 20 more dogs per condition to the present study (80 dogs per condition) compared to the original study (with approx. 60 dogs per condition). In hindsight, however, the effect size in Lonardo et al. (2021), a 19% difference between conditions, might have been too large compared to what we found now, closer to a 10% difference between conditions. This hypothesis is corroborated by lack of evidence for a significant difference between the two studies. However, before data collection, we considered the 19% difference as our minimum effect size of interest (from the perspective of everyday relevance). What seems to be the case is that when dogs can no longer rely on behavioural cues to differentiate between conditions, they are not as sensitive to the same TB-FB experimental manipulation (effect size is smaller), which was exactly the question we were investigating. Assuming an even smaller effect size would have been unreasonable due to a few considerations. First, it would have required a substantially larger sample size (with the observed effect size, a total sample of 420 dogs, 140 per condition, would have yielded a power of 85%), given our one-trial only, between-subjects design. Future research might consider within-subject designs in which participants are tested on multiple trials per condition, but still taking care to minimise likely carry-over effects or testing at least 140 dogs per condition with the present task. Such a feat might be achieved through a Big Team Science project (Alessandroni et al. 2024; ManyDogs Project, Alberghina et al. 2023). Second, assuming a smaller effect size and therefore increasing the sample size would have raised questions about the meaningfulness of a possibly very small difference between conditions, as it was indeed the case in a multi-lab collaboration (ManyDogs Project, Alberghina et al. 2023).

Contrary to the study by Lonardo et al. 2021; no significant breed differences in performance were found in the present study (plots showing the performance of each FCI group in Fig. S2 and of cooperative and independent breeds in Fig. S3). Gácsi et al. (2009) proposed a classification of dog breeds according to their selection pressures to cooperate with humans with (cooperative breeds) or without (independent breeds) maintaining visual contact with their handler. The fact that in the current study we did not find a significant effect of breed group (cooperative vs. independent), neither in interaction with condition nor as a main effect, could be due to the relatively small sample of dogs tested that belonged to independent breeds (*N* = 30). This subsample of independent breeds was however included in the current study to replicate as much as possible the sample of Exp. 1 in Lonardo et al. (2021). At the same time, terriers were not included in the present task, (again, to replicate as much as possible the findings of Experiment 1 of Lonardo et al., where most dogs behaved differently from terriers) and might be a promising candidate for follow-up studies.

In conclusion, the advantages of this novel task are numerous. First, while listening, an experimenter can behave identically between conditions (i.e., the task allows for controlling for behaviour-reading). Second, the experimenter does not have a direct line of gaze to the scene in the TB and FB conditions. Third, potential associative learning explanations (the different sound cues used to manipulate the experimenter’s mental states between TB and FB conditions being sufficient to give a correct response) can be easily controlled for (KSC condition). Fourth, this task does not require additional abilities (such as experience-projection) and instead only tests auditory perspective-taking. Our task tests specifically altercentric perspective taking, it cannot be solved through an egocentric strategy, and it can differentiate between behaviour- and mind-reading in non- or pre-verbal populations. However, our findings indicate that dogs did not significantly differentiate between conditions, and thus we found no evidence that dogs engaged in auditory perspective-taking in this context. Nonetheless, several alternative explanations must be considered. It is possible that dogs interpreted the back-turned communicator as inattentive, or that the lack of behavioural cues made auditory perspective-taking especially challenging. Alternatively, dogs might possess some capacity for altercentric perspective-taking, but the effect may be too subtle to detect with the current methodology or sample size. In line with this, the Bayesian analysis provided some evidence for the predicted effect (that dogs would be more likely to follow the misleading suggestion in FB compared to the other conditions). Overall, while an egocentric strategy may often be sufficient for successful interaction with humans (for example, stealing food silently), further research is needed to clarify whether the apparent limitations in dogs’ performance are task-specific or reflect a broader inability for altercentric perspective-taking, particularly across different sensory modalities. Future studies employing refined methods and larger samples may help determine whether dogs’ abilities in this domain are more limited than in the visual domain, or simply more difficult to detect.

## Supplementary Information

Below is the link to the electronic supplementary material.

Supplementary Material 1

## Data Availability

All datasets, statistical tools and code used for the analyses and plots are available at: https://github.com/lonardol/auditory_false_belief_dogs.
