# Supplementary material for: Is dogs’ tendency to follow human misleading communicative cues influenced by humans’ auditory perspective?
Source: Anim Cogn. 2025 Nov 23;29(1):6. doi: 10.1007/s10071-025-02028-y (PMC12748098; doi:10.1007/s10071-025-02028-y)
Supplement: Supplementary file 1 — Supplementary Material 1 [file 10071_2025_2028_MOESM1_ESM.docx]

**Supplementary Material for:**

**Dogs follow human misleading suggestions more often when the informant has a false belief**

**Lucrezia Lonardo, Victoria Berndl, Christoph J. Völter & Ludwig Huber**

Corresponding author: Lucrezia Lonardo, Messerli Research Institute, University of Veterinary Medicine of Vienna

Email: [lucrezia.lonardo@vetmeduni.ac.at](mailto:lucrezia.lonardo@vetmeduni.ac.at)

Article submitted to: Animal Cognition

**This file includes:**

Supplementary text

Figures S1 to S3

Tables S1 to S5

Legends for Dataset S1

SM References

Other supplementary materials for this manuscript (datasets S1, code and statistical tools used for the analyses and plots) are available at: <https://github.com/lonardol/auditory_false_belief_dogs>

**Supplementary Text**

Description of familiarisation

***Trial 0***

When dogs entered the experimental room, at the beginning of the session, they were let off the leash by their owner and free to explore the room while the owners received some instructions on how to behave during the test in order not to distract their dog. Both experimenters were in the room, together with the dog and owner. The hider held food in her hand. The food used for most dogs was dry kibble (lamb) but whenever a dog was not motivated by dry food, as judged by the experimenters in this phase, or had a food intolerance, sausages (pork) were used, or the owner provided the dog’s usual food. When the dogs had finished exploring the room and approached the hider, the latter started to place food in the open buckets (always one treat hidden at a time, to ensure dogs would not expect multiple treats to be already hidden in the buckets), then she shook the lids to make sure the dog was comfortable enough to eat even during/after the bells noise, and finally she started showing to the dogs how to push the cardboard lid with bells off the buckets. The duration of this phase varied depending on each dog’s boldness and food motivation (approx. between 2 and 25 minutes). Treats were always placed in each bucket sequentially so that overall dogs got the same number of treats from each bucket and did not have time to develop a side preference due to the alternation after each treat.

***Familiarisation phase 1***

The aim of the first familiarisation phase was to make the dog familiar with the hider and with the hiding and displacement of food. At the beginning of each trial, only the dog, the owner and the hider were present in the room. The dog was kept (through collar or harness) by the seated and blindfolded owner at the starting position (marked by a red cross on the floor as shown in Figure 1 A and B), equidistant from both containers and with a full view of the experimenter´s actions (see also Fig. S1). In the first trial of this phase, the “no relocation” trial, food was hidden only in one container (container A). In detail, the hider showed to the dog that she had food and hid it in container A. She closed the container and left the room. Dogs were then released by their owners and were free to approach one of the containers and retrieve its content (if any). If dogs chose the baited container, they moved on to the next trial, otherwise the no-relocation trial was repeated for a maximum of two times in total. In the next trial, dogs additionally witnessed a relocation of the food to container B (“relocation trial”). Immediately (i.e., approximately less than 2 seconds) after closing container A, the hider retrieved the food from there and visibly transferred it into container B. Then, after closing the container, she left again the room. Once more, owners released the dogs after the hider had left and closed the door behind her. If dogs did not move up to 10 seconds from the release, owners were allowed to give verbal encouragement (such as “ok” or “you can go”). After additional 10 s, owners could repeat the encouragement, if necessary. Owners were instructed to refrain from nudging their dog physically. If dogs still did not move, after 30 seconds from the moment of release, we scored the trial as “no choice” and we repeated it for a maximum of 2 trials. If, however, dogs chose the empty container, the hider went back into the room and removed the food from the baited bucket. The incorrect trial was immediately repeated. If dogs made a second mistake they were excluded from the study. If, instead, they chose the baited container, the other type of trial (relocation or no relocation) was repeated again as well. Only dogs that made two correct choices in two consecutive trials (one with relocation, one without relocation) within four trials moved on to the next phase.

***Familiarisation phase 2***

In the second familiarisation phase, we controlled for dogs’ motivation to follow the communicator’s cue to find food even when they had not witnessed the hiding event. The order of relocation and no relocation trials in this condition was counterbalanced across dogs, so for half of the dogs food was hidden in container A and for the other half of the dogs food was hidden in container B in the first trial of this phase and vice versa in the second trial. Hider and communicator entered the room while dog and owner waited outside. The dog and owner stayed outside the room during the baiting of the containers, without visual access to the baiting. However, the dogs saw that hider and communicator went into the room together. Having heard the hiding, the communicator knew the correct location of food. The hider left the room and invited the owner to enter and take the seat in the experimental room. When entering the room, the owner held the dog so that s/he could not approach the containers yet. Upon entering, dogs found the communicator crouched down, in a position equidistant from the two containers, facing the wall (as shown in Fig. 1). Once the dog was in the initial position and the owner was seated and wearing the blindfold again, the communicator suggested to the dog where to look for food. She turned around, approached the baited container, crouched down close to it, picked it up, alternated gaze between it and the dog while saying: “look, this is good, this is very good”. Then she walked back to her starting, neutral, position, this time facing the buckets and looking to the floor, and signaled to the owner to release the dog. In this phase, dogs could rely solely on the communicator’s suggestion to find food above chance level, as they could not witness the hiding procedure. If dogs were correct on both trials, they moved on to the next phase. If not, we used the same procedure and inclusion criteria described above.

***Familiarisation phase 3***

The aim of the third familiarisation phase was twofold; a) to make the dog familiar with the complete hiding and cueing procedure and b) to test, if dogs are attentive and motivated enough to approach the correct (baited) container. Dog and owner started each trial again inside the room, as in familiarization phase 1. Hider and communicator entered the room together. The communicator crouched down facing the wall, in a position equidistant from the two containers. In the “no relocation” trial, the hider showed to the dog that she had food and she hid it in container A. Then, she closed the container before leaving the room. In the “relocation” trial, dogs additionally witnessed a relocation of the food. Immediately after closing container A, the hider retrieved the food from there and visibly transferred it into container B. Then, after closing the box, she left the room. In both trials, once the hider had left the room, the communicator suggested to the dog where to look for food (approach, manipulation of the container, voice and gaze alternation). The communicator then went back to her neutral starting position and said “ok”, to signal to the blindfolded owner that it was time to release the dog. Dogs now had two converging pieces of information about the (final) location of food: their own visual experience of the hiding events, and the communicator’s suggestion. Only dogs that made two correct choices in two consecutive trials (one with relocation, one without relocation) within four trials were subsequently tested in the final test phase.

During the hiding and suggesting procedure, owners were instructed to let the dog freely move their head and body within the reach of their arm. At the end of each trial, owners were allowed to remove the blindfold to recall and catch their dogs and when necessary in order to leave and re-enter the room safely. Owners were made aware of the aim and hypothesis behind the study only after the test.

Scoring and Analyses

***Choice – GLMMs 01, 02, 03 and 04***

Correlation parameters between random slopes and the random intercept of breed were excluded from the model because unidentifiable. We verified the absence of collinearity in the models using variance inflation factors (R package “car”) and that the assumptions of the models were met, in particular that the estimated deviations of intercepts and slopes from the respective common average (BLUPs - Best Linear Unbiased Predictors per level of the random effects) were normally distributed (Baayen, 2008; Harrison et al., 2018).

We assessed the stability of the models with regards to the estimated coefficients and standard deviations by comparing the original estimates with the minimum and maximum of the estimates obtained after case-wise deletion of levels of the random effect (breed; Nieuwenhuis, 2012). Because we were mainly interested in a single test predictor (experimental condition), while the other fixed effects were considered control predictors, the comparison between the full model and null model lacking condition as predictor was implemented by using the R function drop1 (Chambers & Hastie, 1992) with argument 'test' set to "Chisq" to make inferences on the significance of the test predictor. The function drop1 drops each fixed effect from the model (one at a time) and uses a likelihood ratio test to compare the full with the respective reduced model (Barr et al., 2013).

For GLMM 04, age was recoded in years because it was coded in years in the 2021 dataset.

**Figures**


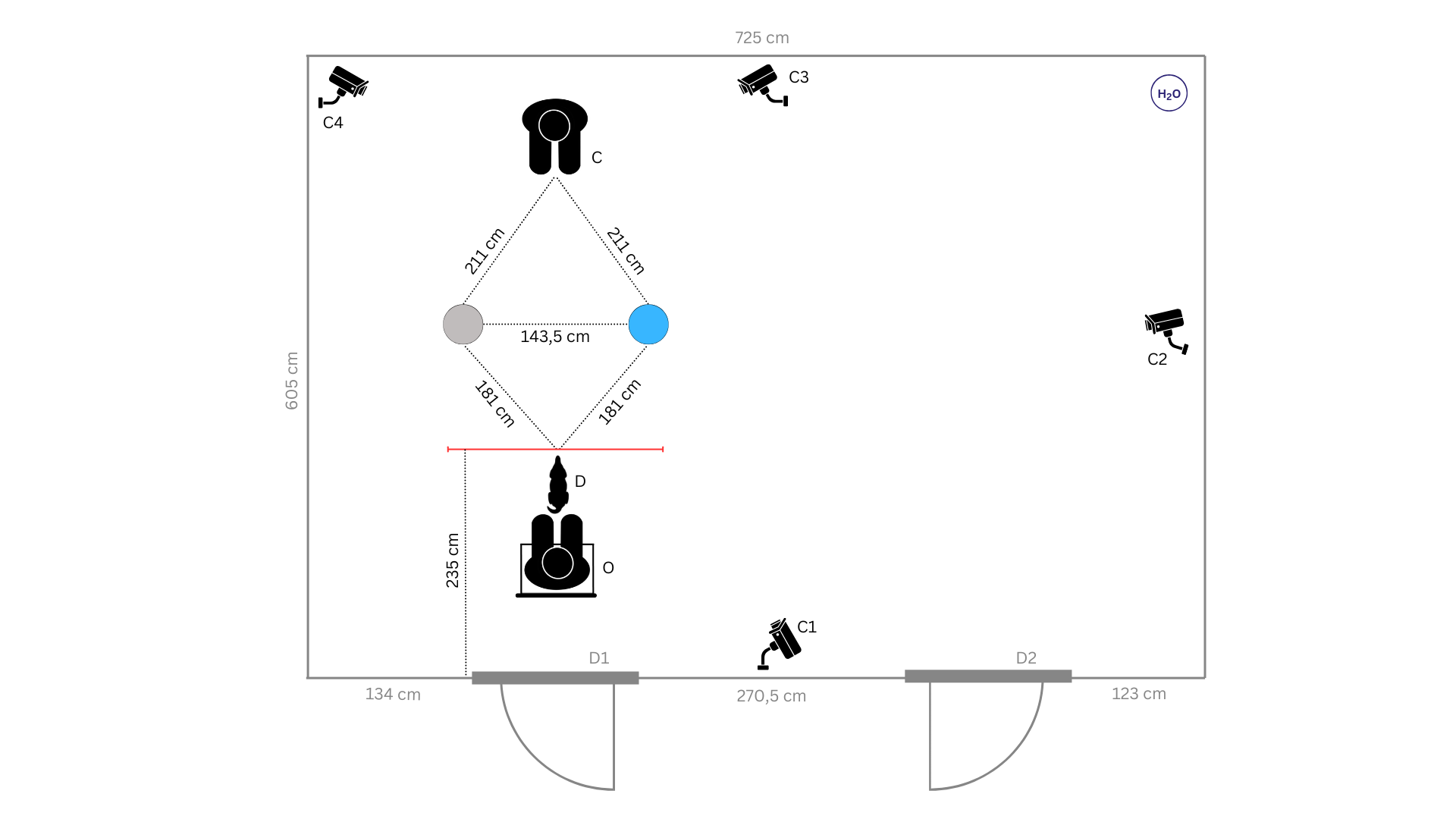


**Fig. S1.** Sketch of experimental room and set-up with measurements. Both dog and communicator were equidistant to the buckets (grey and blue circles). Dogs had unrestricted visual access to the hider´s actions during test. They were held in their starting position, behind the red line and facing the buckets and communicator, by their seated and blindfolded owner. The position of the buckets was kept constant across dogs. Which bucket was baited first was counterbalanced across dogs.

C= communicator; H= hider; D= dog; O= owner; D1= door used by hider and communicator to enter and exit the room throughout the whole experiment. C1, C2, C3 and C4= cameras recording the scene.

**
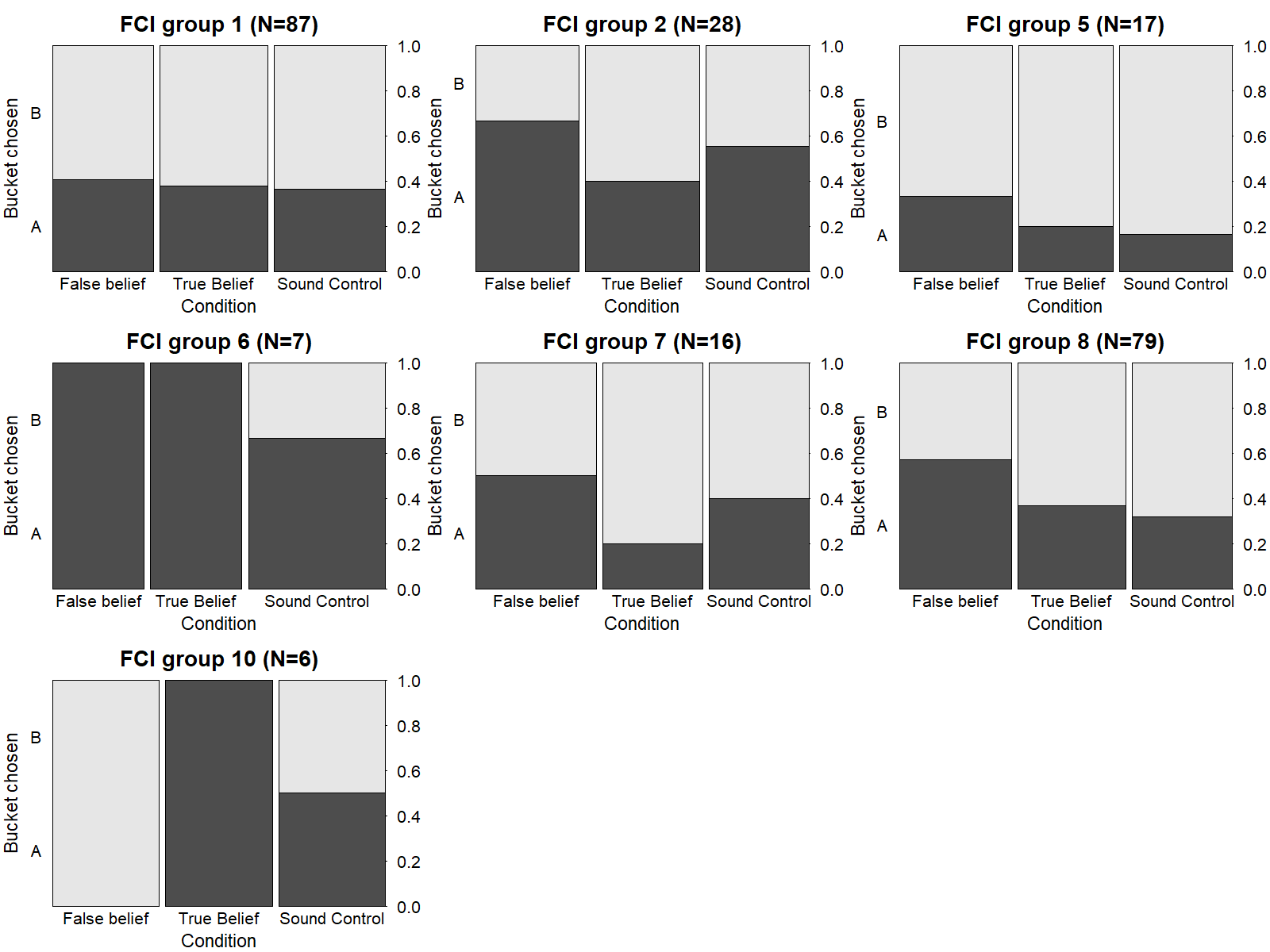
**

**Fig. S2.** The 240 dogs tested in this study could be classified in 7 FCI groups, according to their breed. The plots show dogs’ choices as a function of condition, separately for for each FCI group. The proportion of dogs that chose container A, as suggested by the communicator, is shown in dark grey; the proportion of dogs that chose container B is shown in light grey. Bar width is proportional to the number of individuals tested in each condition.

**
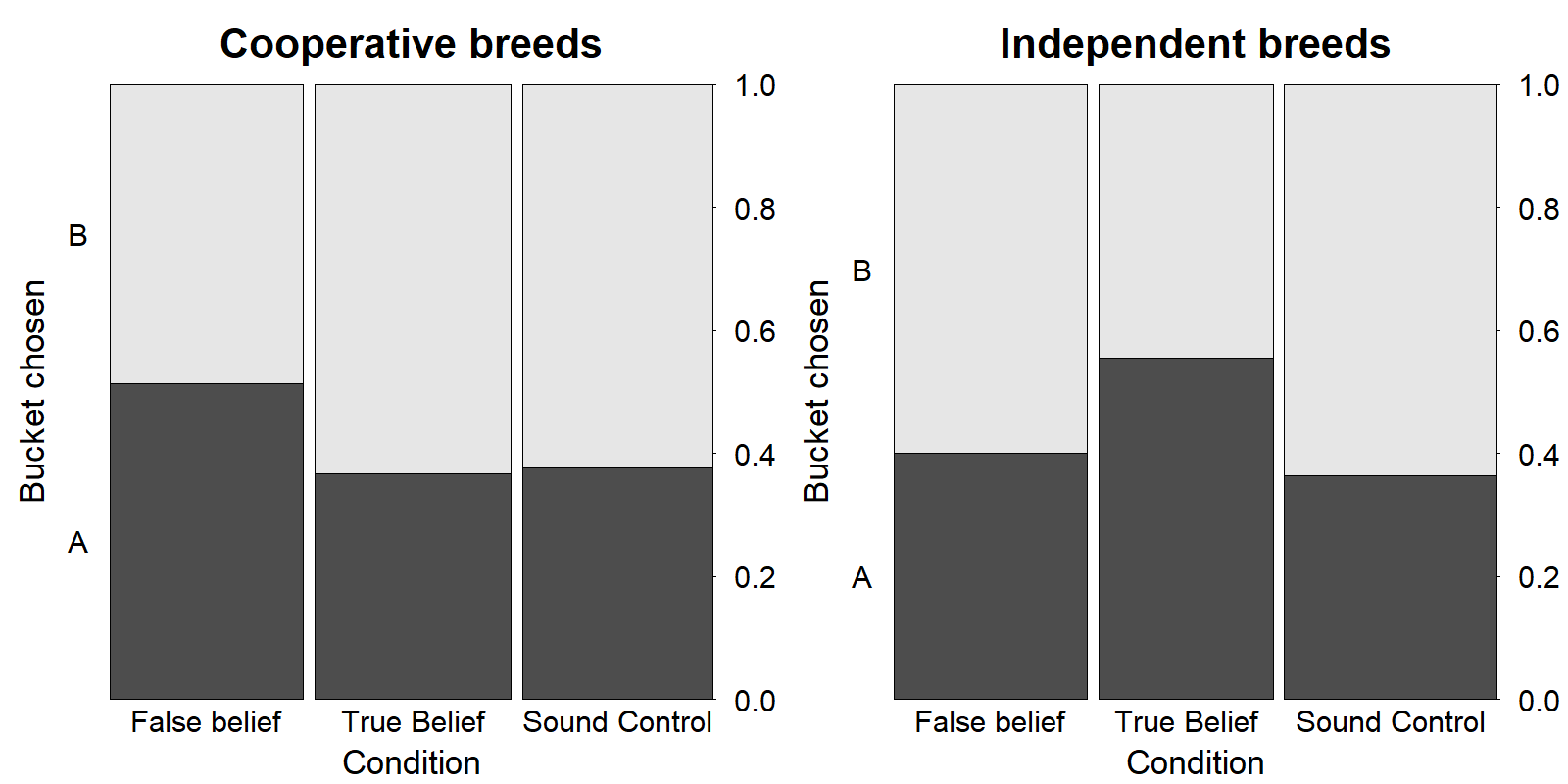
**

**Fig. S3.** Mosaic plot showing dogs’ choices as a function of condition, separately for the two breed groups. Cooperative breeds (FCI groups 1, 2, 7 and 8, in total N=210) are shown on the left and independent breeds (FCI groups 5, 6 and 10, in total N=30) are shown on the right. The plots show dogs’ choice as a function of condition (FB, TB or KSC). The proportion of dogs that chose container B, is represented in the upper part of the plots (light grey bars); the proportion of dogs that chose the empty container A, as suggested by the communicator, is represented in the lower part of the plots (dark grey). Bar width is proportional to the number of individuals tested in each condition. No significant breed group differences were found in dogs’ choices (Table S2).

**Tables**

**Table S1.** Demographic information of the dogs tested in each experimental condition.

|  | Age range | Mean age | Std. Dev. | Nr. females | Nr. males |
| --- | --- | --- | --- | --- | --- |
| FB | 6 - 143 | 59.01 | 35.57 | 44 | 36 |
| TB | 6 - 146 | 60.28 | 38.21 | 43 | 37 |
| KSC | 6 - 155 | 60.23 | 36.22 | 43 | 37 |

Age is reported in months.

FB: False Belief; TB: True Belief; KSC: Knowledge Sound Control.

**Table S2.** Results of GLMM 01 showing no effect of condition on choice (N=240).

|  | Estimate | Std. Error | CI min | Ci max | χ^2^ | df | P | min | max |
| --- | --- | --- | --- | --- | --- | --- | --- | --- | --- |
| Intercept | 0.35 | 0.29 | -0.24 | 1.00 |  |  | ^(1)^ | 0.20 | 0.41 |
| Condition FB^(2)^ | -0.51 | 0.32 | -1.23 | 0.15 |  |  | ^(2)^ | -0.68 | -0.35 |
| Condition TB^(2)^ | -0.05 | 0.33 | -0.75 | 0.67 | 3.03 | 2 | 0.220 | -0.17 | 0.08 |
| Sex^(3)^ | 0.25 | 0.27 | -0.32 | 0.85 | 0.83 | 1 | 0.360 | 0.05 | 0.31 |
| Age^(4)^ | 0.15 | 0.15 | -0.13 | 0.50 | 1.18 | 1 | 0.278 | 0.11 | 0.22 |
| First baited bucket^(5)^ | 0.10 | 0.27 | -0.46 | 0.71 | 0.14 | 1 | 0.710 | 0.04 | 0.26 |

^(1)^ Not shown due to its very limited interpretability

^(2)^ Knowledge Sound Control was the reference category. The likelihood ratio test reported refers to the main effect of Condition.

^(3)^ Females were the reference category

^(4)^ Z-transformed to a mean of 0 and a standard deviation of 1.

^(5)^ Bucket on the dog’s left was the reference category

**Table S3.** Results of GLMM 02 showing no effect of condition on choice (N=240), even when accounting for breed type (cooperative/independent).

|  | Estimate | Std. Error | CI min | CI max | χ^2^ | df | P | min | max |
| --- | --- | --- | --- | --- | --- | --- | --- | --- | --- |
| Intercept | 0.37 | 0.29 | -0.26 | 1.05 |  |  | ^(1)^ | 0.22 | 0.43 |
| Condition FB^(2)^ | -0.51 | 0.32 | -1.23 | 0.16 |  |  | _(2)_ | -0.69 | -0.36 |
| Condition TB^(2)^ | -0.06 | 0.33 | -0.77 | 0.61 | 3.03 | 2 | 0.219 | -0.17 | 0.08 |
| Breed type^(3)^ | -0.11 | 0.40 | -0.97 | 0.77 | 0.07 | 1 | 0.794 | -0.45 | 0.23 |
| Age^(4)^ | 0.15 | 0.14 | -0.12 | 0.46 | 1.23 | 1 | 0.267 | 0.12 | 0.23 |
| Sex^(5)^ | 0.24 | 0.28 | -0.32 | 0.81 | 0.81 | 1 | 0.369 | 0.04 | 0.30 |
| First baited bucket^(6)^ | 0.10 | 0.27 | -0.45 | 0.70 | 0.15 | 1 | 0.699 | 0.04 | 0.27 |

^(1)^ Not shown due to its very limited interpretability

^(2)^ Knowledge Sound Control was the reference category. The likelihood ratio test reported refers to the main effect of Condition.

^(3)^ Cooperative breeds were the reference category

^(4)^ Z-transformed to a mean of 0 and a standard deviation of 1

^(5)^ Females were the reference category

^(6)^ Bucket on the dog’s left was the reference category

**Table S4.** Results of GLMM 03 showing no effect of condition on choice (N=240), even when accounting for dogs’ training (yes/no).

|  | Estimate | Std. Error | CI min | CI max | χ^2^ | df | P | min | max |
| --- | --- | --- | --- | --- | --- | --- | --- | --- | --- |
| Intercept | 0.04 | 0.36 | -0.73 | 0.81 |  |  | ^(1)^ | -0.16 | 0.14 |
| Condition FB^(2)^ | -0.53 | 0.33 | -1.36 | 0.15 |  |  | ^(2)^ | -0.70 | -0.36 |
| Condition TB^(2)^ | -0.08 | 0.33 | -0.81 | 0.62 | 3.09 | 2 | 0.213 | -0.19 | 0.06 |
| Training^(3)^ | 0.45 | 0.31 | -0.17 | 1.12 | 2.13 | 1 | 0.145 | 0.34 | 0.70 |
| Age^(4)^ | 0.14 | 0.14 | -0.14 | 0.51 | 1.14 | 1 | 0.285 | 0.11 | 0.22 |
| Sex^(5)^ | 0.26 | 0.27 | -0.24 | 0.89 | 0.93 | 1 | 0.334 | 0.07 | 0.32 |
| First baited bucket^(6)^ | 0.08 | 0.27 | -0.49 | 0.67 | 0.08 | 1 | 0.773 | 0.02 | 0.22 |

^(1)^ Not shown due to its very limited interpretability

^(2)^ Knowledge Sound Control was the reference category. The likelihood ratio test reported refers to the main effect of Condition.

^(3)^ No training was the reference category

^(4)^ Z-transformed to a mean of 0 and a standard deviation of 1

^(5)^ Females were the reference category

^(6)^ Bucket on the dog’s left was the reference category

**Table S5.** Results of GLMM 04 quantitatively comparing the present results to the 2021 study

|  | Estimate | Std. Error | χ^2^ | df | P | min | max |
| --- | --- | --- | --- | --- | --- | --- | --- |
| Intercept | 0.16 | 0.25 |  |  | ^(1)^ | 0.1 | 0.29 |
| Condition C^(2)^ | 0.72 | 0.25 |  |  | ^(2)^ | 0.58 | 0.82 |
| Condition TB^(2)^ | 0.6 | 0.25 | 9.45 | 2 | .009 | 0.52 | 0.78 |
| Study^(3)^ | -0.29 | 0.21 | 1.9 | 1 | .169 | -0.34 | -0.2 |
| Age^(4)^ | 0.30 | 0.11 | 6.78 | 1 | .009 | 0.25 | 0.37 |
| Sex^(5)^ | 0.29 | 0.21 | 1.86 | 1 | .172 | 0.23 | 0.35 |
| First baited bucket^(5)^ | -0.16 | 0.21 | 0.58 | 1 | .445 | -0.23 | -0.08 |

^(1)^ Not shown due to its very limited interpretability

^(2)^ FB was the reference category. The likelihood ratio test reported refers to the main effect of Condition.

^(3)^ The original (2021) study was the reference category

^(4)^ Z-transformed to a mean of 0 and a standard deviation of 1.

^(5)^ Females were the reference category

^(5)^ Bucket on the dog’s left was the reference category

**Legend for the data file “tested_dogs.csv” (separate file)**. The data file used in the analyses is available at the GitHub repository: <https://github.com/lonardol/auditory_false_belief_dogs>. Age is shown in months.

**Supplementary Material References**

Baayen, R. H. (2008). *Analyzing Linguistic Data: A Practical Introduction to Statistics Using R*. Cambridge University Press. https://doi.org/10.1017/CBO9780511801686

Barr, D. J., Levy, R., Scheepers, C., & Tily, H. J. (2013). Random effects structure for confirmatory hypothesis testing: Keep it maximal. *Journal of Memory and Language*, *68*(3), 255–278. https://doi.org/10.1016/j.jml.2012.11.001

Chambers, J. M., & Hastie, T. J. (1992). Linear models. Chapter 4 of statistical models in S. *Wadsworth & Brooks/Cole*.

Harrison, X. A., Donaldson, L., Correa-Cano, M. E., Evans, J., Fisher, D. N., Goodwin, C., Robinson, B., Hodgson, D. J., & Inger, R. (2018). Best practice in mixed effects modelling and multi-model inference in ecology. *PeerJ Preprints*.

Nieuwenhuis, R. (2012). *Inﬂuence.ME: Tools for Detecting Inﬂuential Data in Mixed Effects Models*. *4*, 11.
